# Editorial: Multi-omics studies and applications in precision medicine

**DOI:** 10.3389/fgene.2022.1034283

**Published:** 2022-10-10

**Authors:** Arivusudar Everad John

**Affiliations:** Mazumdar Shaw Medical Centre, Bengaluru, India

**Keywords:** multi-omics, precision medicine, metabolomics, phosphoproteomics, transcriptomics

In the recent years multi-omics has emerged as a vital approach in discovery studies. With advent of high throughput technologies, it has become feasible for scientists across the globe to perform large scale experiments. Instead of looking at few molecules, Omics studies unleashes the potential of looking at the entire repertoire of genome, transcriptome, epigenome, proteome metabolome or microbiome. Such studies lead to novel discoveries and aids in providing a holistic view of the biological systems. For instance, Zhi-e et al. have applied metabolomics and transcriptomics to delineate the hepatotoxicity in mice models. Using two different approaches, they could pinpoint and emphasize the toxicity associated with the use of Aristolochic Acid I. Mohd et al. employed a phosphoproteomics analysis to decipher the role of a crucial tyrosine kinase CAMKK2 in gastric cancer. They have delineated the entire pathway of CAMKK2 and this study could provide potential novel targets for targeted therapy in gastric cancer.

In the current Omics era, scientists have gone a step further where the publicly available datasets are used to derive deeper insights into the biological problem. In this issue, two groups have actually done a deep dive into the freely available omics datasets to derive meaningful insights of the problem of interest. Kunpeng et al. used TCGA datasets to perform a pan-cancer analysis of the role of 4EBP1 (Eukaryotic translation initiation factor 4E binding protein 1) and its prognostic significance. Similarly, Shizhe et al. used TCGA datasets to develop a prognostic model for prognosis of hepatocellular carcinoma. In summary, multi-omics approaches can be proven useful and serves as a treasure trove in the field of biomarkers.

